# Enhanced antioxidant activity of brown seaweed *Laminaria japonica* by fermentation using isolated *Bacillus subtilis*

**DOI:** 10.1186/s40643-025-00912-6

**Published:** 2025-07-04

**Authors:** Yueh-Hao Ronny Hung, Ya-Han Chang, Hsuan-Ju Lin, Li-Ho Chiang, Hong-Ting Victor Lin

**Affiliations:** 1 Department of Food Science, National Taiwan Ocean University, No. 2, Pei- Ning Road, Keelung, 202 Taiwan, R.O.C.; 2 Center of Excellence for the Oceans, National Taiwan Ocean University, No. 2, Pei-Ning Road, Keelung, 202 Taiwan, R.O.C.

**Keywords:** *Laminaria japonica*, *Bacillus subtilis*, Fermentation, Antioxidant, Polysaccharide, Phenolic compounds

## Abstract

Bioactive compounds from the brown seaweed *Laminaria japonica* have received considerable attention from the food industries to explore their biological activities. However, effectively producing bioactive compounds from seaweed requires further investigation because of the complex composition of seaweed cell walls. In the present study, *Bacillus subtilis* strains were isolated and screened for the production of extracellular hydrolytic enzymes for the fermentation of *L. japonica* to enhance its antioxidant activity. Three *B. subtilis* strains were successfully isolated from commercial natto products, and their hydrolytic abilities were evaluated using fibrinogen, alginate, and cellulose plates. Strain B2 exhibited great application potential in degrading seaweed cell wall polysaccharides compared with other strains. Subsequently, strain B2 was utilized for seaweed fermentation, and chemical composition alterations (including phenolic compounds, total sugar, and reducing sugar) of the fermented broth were analyzed. A high sulfate content was detected in the polysaccharide fraction of the fermented seaweed. Finally, the antioxidant activities (ferrous iron-chelating ability, reducing power, ABTS radical-scavenging ability, and DPPH radical-scavenging ability) of the supernatant, polysaccharide, and polyphenol fractions from the fermented seaweed were assessed and compared with those of the nonfermented groups. Results indicated that fermentation with *B. subtilis* could enhance the antioxidant activity in supernatant, polysaccharide, and polyphenol fractions. Fermentation acted positively on the bioconversion of *L. japonica* extract, thereby improving the antioxidant properties. Overall, this study provides valuable insights into enhancing the antioxidant activities of *L. japonica* through fermentation.

## Highlights

*B. subtilis* natto strains isolated for seaweed fermentation.

Fermentation increased phenols, total sugar, and reducing sugar content.

Sulfate content in polysaccharide fraction increased after fermentation.

Fermentation enhanced antioxidant activities of *L. japonica*.

**Graphical Abstract:**

## Graphical abstract

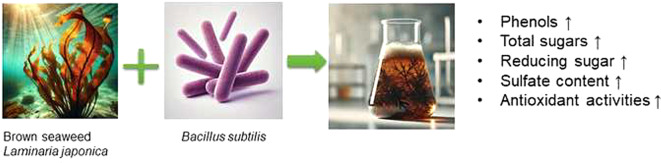

## Introduction

Seaweed is an important food source, providing various nutrients and bioactive compounds. In addition, seaweed is viewed as a potential source for drug developments. For instance, the extracts of red and brown seaweeds were investigated against drug-resistant *Escherichia* (*E.*) *coli* (Lu et al. 2019, 2021). *Laminaria* (*L.*) *japonica* is a brown seaweed that is widely distributed and cultivated in eastern and southern Asia. The bioactive compounds extracted from *L. japonica* exhibit various nutritional benefits, such as antioxidant, anticoagulant, antibacterial activity, and other biological activities (Hwang et al. 2019; Liu et al. 2017; Luan et al. 2021; Wang et al. 2010a, b). The bioactive compounds derived from *L. japonica* are categorized into two major groups. For example, alginate, fucoidan, and laminarian are known bioactive polysaccharides (Holdt and Kraan 2011), and phlorotannin is a group of bioactive phenolic compounds (Li et al. 2011). Moreover, the protein, lipid, pigment, vitamin, and mineral fractions of seaweeds have shown great potential for food and pharmaceutical applications (Pradhan et al. 2022). Thus, the identification and extraction of bioactive compounds from *L. japonica* have received considerable attention because of their potential implication for various biological processes. However, effectively acquiring bioactive compounds from seaweeds is hindered by the complexity of the algal cell wall and related cell-wall-bound compounds (Wijesinghe and Jeon 2012a, b). For example, although hot-water extraction is a common method to obtain seaweed polysaccharides, the loss of bioactivity from the extracted polysaccharides may hinder its applications (Li et al. 2022).

Therefore, exploring an efficient extraction method is essential to address the structural barrier of algal cell walls. Extraction methods can affect the extraction efficiency of bioactive compounds and the performance of biological activity. For example, the molecular weight distribution, monosaccharide composition, and sulfate content of *L. japonica* polysaccharides were remarkably affected by the extraction methods (Gao et al. 2017; Yin et al. 2021). According to Sun et al. (2018), acid extraction or alkaline extraction could reduce the glycosidic linkage more effectively than hot-water extraction, leading to a low viscosity and low average molecular weight in the extracted polysaccharides. Apart from chemical reagent treatments, ultrasonic and microwave techniques can disrupt algal cell walls and allow the extraction of bioactive compounds. Yin et al. (2021) demonstrated that ultrasound-assisted extraction obtained a higher yield and sulfate content in *L. japonica* polysaccharides than hot-water extraction and acid-assisted extraction. Yuan and Macquarrie (2015) indicated that microwave-assisted extraction takes shorter time to extract fucoidan, and this method maintains similar biological activities compared with the conventional acid extraction approach. In developing an efficient extraction process, retaining or enhancing the biological activity of the functional compound from the process is critical. A nondestructive extraction approach is a potential pathway to acquire bioactive compounds from seaweed.

Enzyme-assisted extraction is a nondestructive and mild extraction approach that effectively degrades the algal cell wall and releases bioactive compounds from seaweeds (Wijesinghe and Jeon 2012a, b). The selective characteristic of enzymes promote specific degradation, leading to a high product yield and purity, while minimizing by-product formation. Wang et al. (2010a, b) improved the extraction yield of the red seaweed *Palmaria palmata* by using a commercial enzyme (Umamizyme; endo- and exo-peptidase complex), as compared with water extraction. In addition, the enzyme treatment improved antioxidant activities of the seaweed extract, such as DPPH radical-scavenging activity (Wang et al. 2010a, b), the measurement of the ability of antioxidants to donate hydrogen atoms or single-electron to neutralize DPPH free radicals. However, with regard to industrial-scale production, adding multiple enzymes in each extraction batch may not be economically efficient. Thus, fermentation using microorganisms as biocatalysts provides a potential pathway to perform multiple-enzyme reactions in reproducible batches. Given the capacity of bioconversion, fermentation can play an alternative role in releasing bioactive compounds and enhancing their biological properties. In the past few years, fermentation has received considerable attention because of its contribution to the bioconversion of plant-based biomass for the development of valuable and functional compounds (Hur et al. 2014; Zhao et al. 2021). Although fermentation is primarily applied to biofuel production from seaweed, there is increasing interest in their use for food and nutraceutical product development (Reboleira et al. 2021).

In this study, the effects of fermentation on the brown seaweed *L. japonica* were investigated, and its antioxidant features were explored to promote the development of new products and to broaden the application potential of seaweed in the food sectors. *Bacillus subtilis*, a generally recognized as safe (GRAS) microorganism, was isolated from commercial natto products and selected for multiple extracellular hydrolytic enzyme production and seaweed fermentation. The antioxidant activity of the fermented product was evaluated on the basis of its ferrous iron-chelating ability, reducing power, DPPH-scavenging ability, and ABTS-scavenging ability, and it was compared with that of the nonfermented one. Overall, the result of this study will provide valuable insights into the bioconversion of seaweed for bioactive compound production.

## Materials and methods

### Seaweed and chemicals

Brown seaweed, *Laminaria* (*L.*) *japonica*, was purchased at a local market in Quemoy, Taiwan. *L. japonica* was washed and dried with cold air. The dried seaweed was ground and sieved particle size for 0.25 mm. The fine seaweed powder was stored in a desiccator until use.

Tryptic soy broth (TSB) was purchased from Becton, Dickinson and Company (Sparks, Nevada, USA). Glycerol, ammonium sulfate [(NH_4_)_2_SO_4_], potassium phosphate dibasic (K_2_HPO_4_) were purchased from Bionovas (Toronto, Ontario, Canada). Fibrinogen, thrombin, sodium alginate, magnesium sulfate (MgSO_4_), sodium chloride (NaCl), sodium rhodizonate (Na_2_C_6_O_6_), trichloroacetic acid, 2,2-azino-bis (3-ethylbenzothiazoline-6-sulfonate radical) (ABTS•+), 1, 1-diphenyl-2-picryl-hydrazil (DPPH) were purchased from Sigma-Aldrich (St. Louis, Missouri, USA). Agar, yeast extract, and glucose were purchased from Formedium (Hunstanton, Norfolk, UK) Agarose was purchased from Bioman (Taipei, Taiwan). Carboxymethyl cellulose (CMC) was purchased from Acros (Morris Plains, New Jersey, USA). Ferrous chloride was purchased from Merck (Darmstadt, Frankfurter, USA). All other chemicals were of analytical grade.

### Isolation of *Bacillus subtilis* from nattos

The isolation of *Bacillus subtilis* from natto was followed by the method of Lin et al. (2016). Three *Bacillus subtilis* was isolated from three commercial natto products, respectively. 25 g of commercial natto was mixed with 225 mL sterilized water by using a high-speed homogenizer for 1 min. The mixture was diluted serially, and the 0.1 mL of dilution was used for spreading cultures on a plate count agar (PCA) at 37 °C for 24 h. An isolated colony was picked up and streaked on a new PCA, and the plate was cultured at 37 °C for 24 h. One isolated colony from the streak plate was selected and grew cells in 10 mL TSB at 37 °C/180 rpm for enzyme assay and fermentation. The remaining culture in TSB was mixed with 80% glycerol for glycerol stocks, stored at -80 °C. To assess potential hydrolytic abilities from the isolated *B. subtilis*, three hydrolases related to the brown seaweed cell wall were investigated. The control strain *B. subtilis* BCRC 14,715 was purchased from Bioresource Collection and Research Center (Hsinchu, Taiwan).

### Fibrinolytic enzyme assay

Fibrinolytic activity from three isolated *B. subtilis* was carried out by fibrin plate assay, described by Ko et al. (2004) with modifications. 20 mL of fibrinogen solution [0.6% (w/v) fibrinogen in 50 mM phosphate buffer (pH 7.4)] was mixed with 20 mL of agarose solution (1.25%, w/v) and then mixed with 0.4 mL of thrombin solution [10 unit/mL in 50 mM phosphate buffer (pH 7.4)]. The mixed solution underwent filter sterilization before being dispensed into Petri dishes containing sterilized Oxford cups. Once the plates solidified, the Oxford cups were removed, the plates were wrapped with parafilm and stored at 4 °C until use.

Isolated *B. subtilis* strains were grown in TSB at 37 °C for 6 h. Then 100 µL of *B. subtilis* culture was added in each hole on the fibrin plate and incubated at 37 °C for 18–24 h. Hydrolyzed fibrinogen showed clear zone on the agar plate. Diameter of clear zone was measured to compare the fibrinolytic enzymes activity between strains.

### Alginate lyase assay

Enzyme assay for alginate lyase was carried out as previously reported with some modifications (Wang et al. 2017). The alginate agar medium was composed of 0.5% (w/v) sodium alginate, 0.5% (w/v) (NH_4_)_2_SO_4_, 0.2% (w/v) K_2_HPO_4_, 0.1% (w/v) MgSO_4_, and 2% (w/v) agar, and the medium was sterilized by autoclave (121 °C/20 min). After the sterilization, the alginate agar medium was poured into Petri dishes containing sterilized Oxford cups. Once the plates solidified, the Oxford cups were removed, the plates were wrapped with parafilm and stored at 4 °C until use.

Isolated *B. subtilis* strains were grown in TSB at 37 °C for 6 h. Then 60 µL of *B. subtilis* culture was added in each hole on the fibrin plate and incubated at 37 °C for 24 h. Gram’s iodine solution was added to the surface of the agar plate and left to react for 10 min, during which the iodine combined with alginate to form a blue-black complex. After the reaction, distilled water was used to wash off the dye for clearer observation. A yellow zone appeared where alginate had been hydrolyzed, preventing the iodine from binding and forming the complex. Diameter of yellow zone was measured to compare the alginate lyase activity between strains.

### Cellulase assay

Enzyme assay for cellulase was carried out as previously reported with some modifications (Guo et al. 2016). The cellulose agar medium was prepared by 0.5% (w/v) CMC, 0.5%(w/v) (NH_4_)_2_SO_4_, 0.2% (w/v) K_2_HPO_4_, 0.1% (w/v) MgSO_4_, and 2% (w/v) agar. After the sterilization, the cellulose agar medium was poured into Petri dishes containing sterilized Oxford cups. Once the plates solidified, the Oxford cups were removed, the plates were wrapped with parafilm and stored at 4 °C until use.

Isolated *B. subtilis* strains were grown in TSB at 37 °C for 6 h. Then 60 µL of *B. subtilis* culture was added in each hole on the fibrin plate and incubated at 37 °C for 24 h. Congo red was added to the surface of the agar plate and left to react for 10 min, during which the dye combined with cellulose to form a red complex. After the reaction, 1 M NaCl was used to wash off the dye for clearer observation. An orange zone appeared where cellulose had been hydrolyzed, preventing the Congo red from binding and forming the complex. Diameter of orange zone was measured to compare the cellulase activity between strains.

### Seaweed fermentation

*L. japonica* medium was prepared as fellows: 5% (w/v) *L. japonica* suspension was supplied with 2% (w/v) yeast extract, and the medium was adjusted to pH 6.0. Fermentation conditions were followed by Lin et al. (2016). 1% (v/v) *B. subtilis* natto B2 was inoculated into 250 mL of the seaweed medium, and the fermentation was conducted at 37 °C with 150 rpm for 72 h. The fermented broth was centrifuged by 7,000 rpm for 20 min at 4 °C. The fermented supernatant was collected for following experiments. On the other hand, 250 mL of *L. japonica* medium without microbial inoculation was viewed as the control (non-fermented group) and used to compare with the fermentation group. The extracted supernatant was collected by the same centrifugation conditions.

### Chemical composition analysis

Compositions of the fermented and non-fermented broths were analyzed as fellows. Measurement of total phenolic compounds was carried out as previously reported with some modifications (Oktay et al. 2003). 1 mL of sample mixed with 2.5 mL Folin-Ciocalteu’s phenol reagent (10-times dilution). After 3 min, 3 mL of 2% sodium carbonate (Na_2_CO_3_) was added. The mixture was shaking for 2 h without exposing to light. The absorbance was measured at 760 nm, and gallic acid was used as the standards (20–100 mg/L) for quantification. Total sugar content was measured by phenol-sulfuric acid method according to Dubois et al. (1956) with some modifications. 0.4 mL of sample was mixed with 0.2 mL of 5% phenol and then 1 mL of concentrated sulfuric acid was added. After reacting 20 min, the absorbance was measured at 490 nm. Reducing sugar content was quantified by dinitrosalicylic acid (DNS) reagent, described by Miller (1959). Glucose was used as the standards (0–100 mg/mL) for determining both total sugar and reducing sugar contents in the fermented and non-fermented broths.

### Extraction of polysaccharides and polyphenols

Polysaccharide and polyphenol are extracted from the fermented and non-fermented broths, respectively. Polysaccharide extraction was followed by the report of Hentati et al. (2018) with modifications. The supernatant of the fermented broth or the non-fermented broth was mixed with 3 volumes of 95% ethanol (4 °C), centrifuged (8,000 rpm for 10 min at 4 °C) to collect the polysaccharide precipitate, and then the precipitate was freeze-dried for assessments of biological activities (Sect. “Chemical composition analysis”.) and sulfate content (Sect. “Seaweed fermentation”.).

Polyphenol extraction was followed by Wijesinghe et al. (2012) with modifications. 2 g of the supernatant powder was mixed with 100 mL of 80% ethanol and kept in a shaking incubator at room temperature with 120 rpm for 24 h. The mixture was filtered, and then the filtrate was evaporated under vacuum at 40 °C and followed by freeze-drying to obtain the polyphenol extracts.

### Sulfate content analysis

The sulfate content of the polysaccharides gained from the fermented or non-fermented broths was determined by the method of Terho and Hartiala (1971) with modifications. The polysaccharide sample was pretreated by following steps. 0.5 mL of sample was mixed with 0.5 mL of dilute hydrochloric acid (1 M), and the mixture was incubated at 100 °C for 1 h to degrade the polysaccharide. Centrifugal evaporator was used (1,500 rpm) to dry the hydrolysates. The dried content was dissolved in 0.5 mL of Mill-Q water and mixed with 2 mL of 95% ethanol, 1 mL of the barium chloride reagent, and 1.5 mL of the sodium rhodizonate reagent. The mixture was incubated in dark environment for 20 min prior to absorbance measurement at 520 nm. Sodium sulfate (Na_2_SO_4_) was used as the standards (0–40 mg/mL) for quantification of sulfate content from the polysaccharide samples. The barium chloride reagent was prepared by mixing 10 mL of acetic acid (CH_3_COOH; 2 M), 2 mL of barium chloride (BaCl_2_; 0.005 M), and 8 mL of sodium bicarbonate (NaHCO_3_; 0.02 M), and then adjusting the volume to 100 mL with absolute alcohol. The sodium rhodizonate reagent was prepared as follows. 5 mg of Na_2_C_6_O_6_ was dissolved in 20 mL of Milli-Q water and mixed with 100 mg of ascorbic acid. The mixture was then brought to a final volume of 100 mL with absolute alcohol and left to stand in a dark environment for 30 min.

### Antioxidant activity

#### Ferrous iron chelating activity

Ferrous iron chelating activity of the fermented seaweed and non-fermented seaweed was determined by the method of Kuda et al. (2005) with modifications. Supernatant, polysaccharides, and polyphenols fractions of the fermented seaweed or non-fermented seaweed was investigated individually. 0.1 mL of sample was mixed with 0.1 mL distilled water and 0.025 mL of ferrous chloride (0.5 mM), and the absorbance of the mixture was measured at 550 nm (A_1_). 0.025 mL of ferrozine (2.5 mM) was added into the above mixture to react for 20 min. After the reaction, the absorbance was measured at 550 nm (A_2_). The ferrous iron chelating activity was determined by the following Eq. (1):

1 $$\eqalign{ Chelating\, & activity{\rm{ }}\left( \% \right){\rm{ }} \cr & = \left[ {1 - {{(A2 - A1)} \over {(B2 - B1)}}} \right] \times 100 \cr} $$

where A is the absorbance of the sample at 550 nm, while B is the absorbance of the blank (distilled water) at 550 nm.

#### Reducing power

Reducing power of the fermented seaweed and non-fermented seaweed was determined by the method of Wang et al. (2008) with modifications. 0.2 mL of sample was mixed with 0.2 mL of phosphate buffer (200 mM, pH 7.2) and 0.2 mL of 1% potassium ferricyanide (K_3_Fe(CN)_6_) and incubated at 50 °C water batch for 20 min. After cooling, 0.2 mL of 10% trichloroacetic acid was added to the mixture to terminate the reaction. The mixture was centrifuged by 3,000$\:\times\:$g for 10 min. 0.125 mL of supernatant was then mixed with 0.125 mL of distilled water and 0.025 mL of 0.1% ferric chloride (FeCl_3_). The absorbance was measured at 700 nm. Ascorbic acid was used as the standards (0–100 mg/L) for quantification of reducing power.

#### ABTS radical-scavenging activity

ABTS (2,2′-azino-bis-3-ethylbenzthiazoline-6-sulphonic acid) radical-scavenging activity of the fermented seaweed and non-fermented seaweed was evaluated by the method of Chiboub et al. (2017) with modifications. The ABTS radical cation was prepared by mixing 7 mM ABTS solution at pH 7.4 (5 mM NaH_2_PO_4_, 5 mM Na_2_HPO_4,_ and 154 mM NaCl) with 2.45 mM potassium persulphate (K_2_S_2_O_8_) in the volume ratio of 1:1. 0.02 mL of sample was mixed with 0.18 mL of ABTS radical cation (1.8 mM), and the absorbance was measured at 734 nm. Trolox was used as the standards (0.025–0.25 mM) for quantification of ABTS radical-scavenging activity (Trolox equivalent antioxidant capacity, TEAC).

### DPPH radical-scavenging activity

DPPH (1,1-diphenyl-2-picrylhydrazyl) radical-scavenging activity of the fermented seaweed and non-fermented seaweed was determined by the procedure of Oktay et al. (2003) with modifications. 0.1 mL of sample was mixed with 0.5 mL of DPPH (100 µM; prepared in 95% ethanol). The mixture was incubated in the dark environment at room temperature for 40 min prior to the absorbance measurement at 517 nm. The DPPH radical-scavenging activity was determined by the following Eq. (2):

2 $$\eqalign{ DPPH\,radical - & scavenging\,activity{\rm{ }}\left( \% \right) \cr & = \left[ {1 - {A \over B}} \right] \times 100 \cr} $$

where A is the absorbance of the sample at 517 nm, B is the absorbance of the blank (distilled water) at 517 nm.

### Data analysis

Data analysis was done by SPSS Version 12.0 (SPSS Inc., Chicago, IL, USA) and expressed as mean$\:\pm\:$standard deviation. All measurements were replicated three times. The data were analyzed by one-way analysis (ANOVA), and Duncan’s multiple range test was applied to the result, where *p* < 0.05 indicates the significant differences.

## Results and discussion

### Isolation and selection of *B. subtilis* natto

**Fig. 1:**
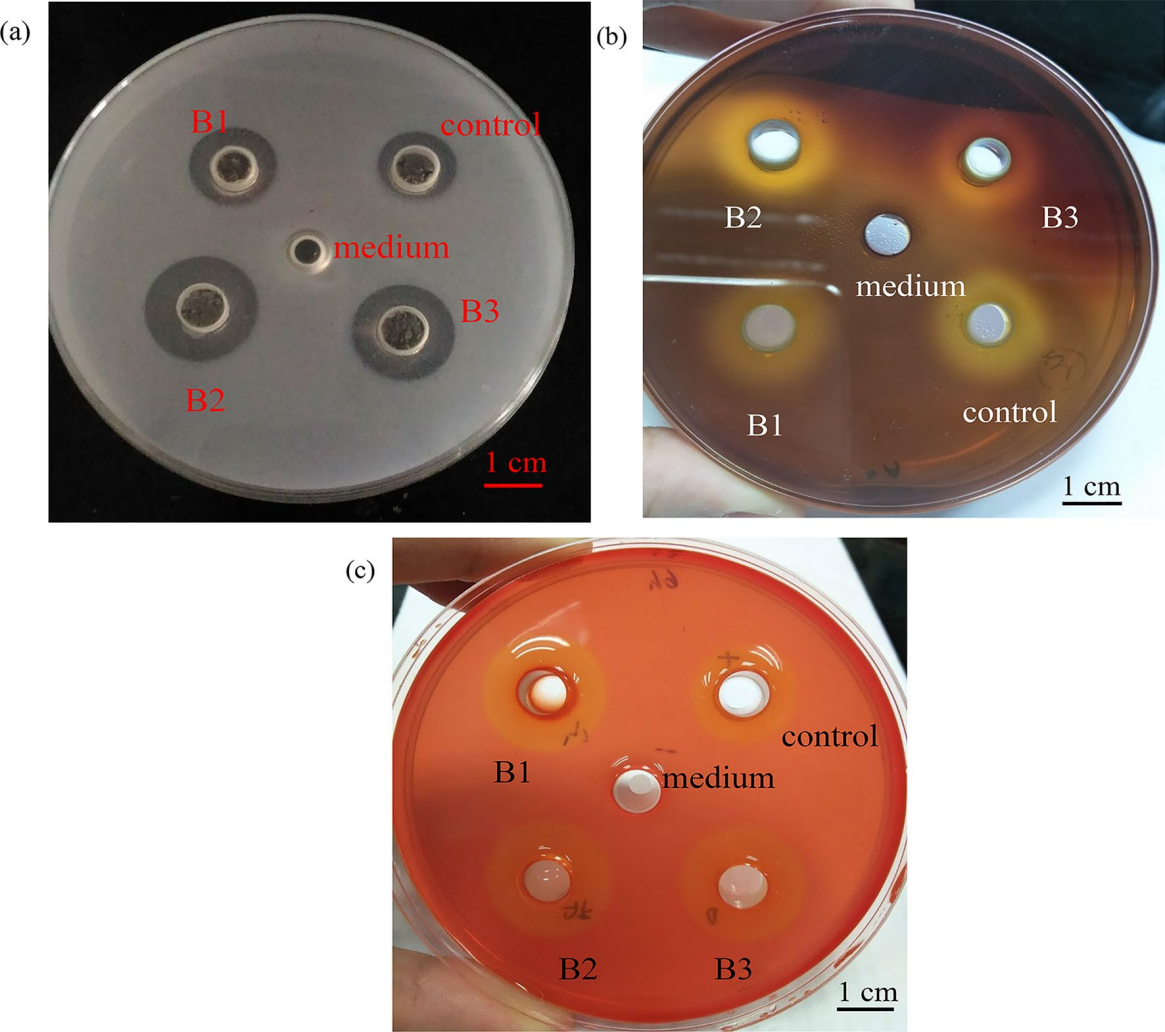
Occurrence of clear zones on the (**a**) fibrin plate, (**b**) alginate plate, and (**c**) cellulose plate applied with the medium and the cultures of control B. subtilis and isolates B1,B2, and B3. (1a) B. subtilis strain were grown in TSB at 37 °C for 6 h prior to the analysis. 100 µL of all culture and plain TSB was inoculated into the wells and the plates were incubated at 37 °C for 18–24 h before the observation of clear zones. (1b and 1c) B. subtilis strain were grown in TSB at 37 °C for 6 h prior to the analysis. 60 µL of all culture and plain TSB was inoculated into the wells and the plates were incubated at 30 °C for 24 h before the observation of clear zones

Three *B. subtilis* natto (B1, B2, and B3) were isolated from commercial natto products. Hydrolytic enzyme production was assessed by using agar plates. Figure 1 shows the occurrence of clear zones on the fibrin plate (1a), alginate plate (1b), and cellulose plate (1c) with the control strain *B. subtilis* BCRC 14,715 and the three isolates B1, B2, and B3. The medium was used as a negative control, showing no clear zone on all agar plates. On the contrary, all three isolates and the control *B. subtilis* could produce enzymes, demonstrating the hydrolyzed zones on the fibrin plate (1a), alginate plate (1b), and cellulose plate (1c). Alginate and cellulose are two common cell wall polysaccharides present in brown seaweeds (Rioux and Turgeon 2015). In particular, alginate accounts for 10–47% of the dry weight of brown seaweed biomass (Holdt and Kraan 2011). Sun et al. (2020) reported that the combination of alginate lyase and cellulase greatly facilitated the hydrolysis of the cell wall polysaccharides of *L. japonica*. Compared with the negative control (Fig. 1), the three isolates (B1–B3) could degrade alginate and cellulose on the agar plates, demonstrating the potential degradation capability of *L. japonica* during fermentation. In addition, *B. subtilis* natto is known for its ability to produce nattokinase, a fibrinolytic enzyme that can degrade fibrin in blood clots (Dabbagh et al. 2014). The isolates B2 and B3 demonstrated a larger clear zone on the fibrin plate than the B1 isolate and control strain. Fibrinolytic activity can positively promote the application of fermented products in the food and pharmaceuticals industries (Dabbagh et al. 2014). While fermentation offers various functional advantages, it also has possible negative effects. Toxic by-products, such as biogenic amines and nitrosamines, are likely formed during fermentation. The precursors of those by-products can be found in vegetables, milk, meat, or other food sources (Wang et al. 2023). Monitoring and controlling the fermentation process becomes more critical in the food industry. In addition to the precursor source, fermentation strain needs to be more reliable. For example, fermentation containing Gram-negative bacteria may increase the risk of endotoxin occurrence in fermented food (Adekoya et al. 2019). In this study, we applied *B. subtilis* to seaweed fermentation, which strain is a Gram-positive bacterium and considered as a GRAS organism. In this case, we can minimize the risk of producing toxic by-products from the fermentation strain. The size of the clear zone (mm) for each plate is shown in Table 1. Although *B. subtilis* B2 and B3 had similar performance to degrade fibrinogen, alginate, and cellulose, *B. subtilis* B2 showed a larger mean value in the size of clear zone and a relatively strong clear zone on the agar plates (Fig. 1). In addition, standard deviations of the clear zone occurrence were smaller when using *B. subtilis* B2, showing relative stability in the enzyme secretions compared to *B. subtilis* B3. Thus, we consider *B. subtilis* B2 as the most promising strain for seaweed fermentation and select *B. subtilis* B2 to ferment the brown seaweed *L. japonica*.

**Table 1:** Clear zone formation by *B. subtilis* isolates from various Natto products on differential medium

Sample	Occurrence of clear zone (mm)		
	Fibrinogen	Alginate	Cellulose
Medium	N.D.	N.D.	N.D.
Control	12.3 ± 0.6^b^	17.0 ± 1.0^a^	16.3 ± 1.2^a^
*B. subtilis* natto B-1	15.0 ± 1.7^b^	17.3 ± 1.2^a^	18.0 ± 4.6^a^
*B. subtilis* natto B-2	17.6 ± 0.6^a^	18.0 ± 0.1^a^	21.0 ± 1.0^a^
*B. subtilis* natto B-3	16.0 ± 1.7^a^	17.3 ± 1.2^a^	21.0 ± 0.1^a^

N.D., not detected

Data are expressed as mean ± SD (*n* = 3)

Different letters in each column show significant differences (*p* < 0.05)

### Chemical composition of the fermented and nonfermented seaweed extracts

*Laminaria* sp. dry biomass composition has been extensively studied and documented to contain several valuable components in varying proportions: approximately 17–46% alginate, which serves as a structural polysaccharide; 0.5–30% laminarin, a central energy metabolite; 2–25% mannitol, a sugar alcohol that acts as an osmolyte and energy reserve; and 3–21% protein, which contributes to the algae’s cellular functions and nutritional value (Becker et al. 2020; Holdt and Kraan 2011). IPolysaccharides are a primary group of bioactive compounds from *L. japonica*, and they have various biological activities, such as antioxidant, anticoagulant, and anticancer (Li et al. 2022). In addition, compared with green and red seaweeds, phenolic compounds, such as phlorotannins, are a unique group of bioactive compounds predominantly found in brown seaweeds (Holdt and Kraan 2011). In the present study, the contents of phenolic compounds, total sugar, and reducing sugar derived from fermented and nonfermented *L. japonica* were assessed (Table 2). During the incubation period, water-soluble compounds from seaweed are likely to be released into water (Chen et al. 2012). However, more water-soluble compounds are released by fermenting the seaweed. The contents of phenolic compounds, total sugar, and reducing sugar in the fermented seaweed suspension were 204.58, 28.91, and 11.58 mg/mL, respectively, which were significantly higher than those in the nonfermented seaweed suspension (176.71, 21.86, and 5.28 mg/mL, respectively). Farvin and Jacobsen (2013) pointed out that higher total phenolic contents correlate to antioxidant activities of seaweed extracts. Assessing the profile of phenolic compounds from seaweed extracts can further help to understand the specific antioxidant mechanism. For example, relatively high levels of caffeic acid, gentisic acid, gallic acid, and vanillic acid in the extracts contributed to positive antioxidant properties in the brown seaweed *Fucus* species (Farvin and Jacobsen 2013). We currently found out that the content of phenolic compounds was higher after fermentation process. In the future of work, evaluating the profile of phenolic compounds in the fermented *L. japonica* can provide further insights into the antioxidant properties and mechanisms.

In addition, a higher sulfate content (10.83 mg/mL) was detected in the polysaccharide extract after fermentation, whereas 6.39 mg/mL of sulfate content was found in the nonfermented polysaccharide extract. Sulfate content of polysaccharides and total phenolic contents are critical variables to evaluate the potential of biological activity for bioactive compounds (Farvin and Jacobsen 2013; Wang et al. 2008). For example, Rocha de Souza et al. (2007) indicated that sulfate content of seaweed polysaccharides showed a positive correlation to the antioxidant activity. Hu et al. (2010) also reported that sulfated polysaccharides showed greater antioxidant activity than de-sulfated polysaccharides. The electron-withdrawing sulfate groups may increase the electrophilicity of adjacent sugar hydroxyls or ring carbons, facilitating electron transfer from reactive free radicals (Tsiapali et al. 2001). It is also suggested that the presence of sulfate groups can enhance metal ion chelating ability. Chelation of redox-active metal ions (e.g., Fe^2+^, Cu^2+^) by sulfate-containing sites can inhibit Fenton-like reactions, indirectly reducing ROS production (Costa et al. 2010; Wang et al. 2010a, b). Higher sulfation may alter the conformation or branching of the polysaccharide, increasing the exposure or density of reactive sites for radical scavenging (Ale et al. 2011). Based on the structure of fucoidans, sulfation can alter the three-dimensional structure of polysaccharide, which may affect the biological activities of polysaccharides (Cho et al. 2010; Wijesinghe and Jeon 2012a, b). In the present study, we observed that fermentation improved sulfate content of polysaccharides and total phenolic contents as the *B. subtilis* B2 performed a positive bioconversion ability. This indicates that our fermented samples have great potential for biological properties. This positive outcome achieved via fermentation was consistent with the report of Shobharani et al. (2013), that is, fermenting *Sargassum* sp. (brown seaweed) with the isolated lactic acid bacteria *Enterococcus faecium* (P1-2CB-w1) could improve the contents of total phenolic compounds, total sugar, and reducing sugar. As fermentation is a bioconversion process that involves various microbial metabolic reactions, multiple enzymes produced from microorganisms can breakdown the algal cell wall and enhance the release of bioactive compounds (Eom et al. 2011; Rafiquzzaman et al. 2015). In our preliminary investigation, the extraction yield of polysaccharides or polyphenols derived from the fermented seaweed suspension showed no significant difference from that of polysaccharides or polyphenols derived from nonfermented seaweed suspension. Fermentation is a microbial bioconversion process that can change the composition of bioactive compounds but does not necessarily affect the extraction yield (Sadh et al. 2018). Extraction yield is varied by fermentation conditions, such as cultivation medium, temperature, pH, or other environmental variables (Hur et al. 2014). However, more phenolic compounds, total sugar, and reducing sugar were detected from the fermented seaweed (Table 2), indicating that the number of compounds per unit of the fermented sample was higher than that of the nonfermented sample. To further evaluate the nutraceutical potential of the fermented seaweed, their antioxidant activities were assessed under various in vitro assays, including ferrous iron-chelating activity, reducing power, ABTS radical-scavenging activity, and DPPH radical-scavenging activity.

**Table 2:** Chemical composition of fermented and non-fermented *L. japonica* suspension

Process	L. japonica suspension		
	Phenolic compounds (mg/L)	Total sugar (mg/mL)	Reducing sugar (mg/mL)
Fermented	204.58 ± 11.90^a^	28.91 ± 2.70^a^	11.58 ± 0.01^a^
Non-fermented	176.71 ± 6.07^b^	21.86 ± 2.19^b^	5.28 ± 0.01^b^

Values are presented as mean ± standard deviation (*n* = 3)

Different superscript letters within the same column indicate significant differences (*p* < 0.05) between processes

### Ferrous iron-chelating activity

**Fig. 2:**
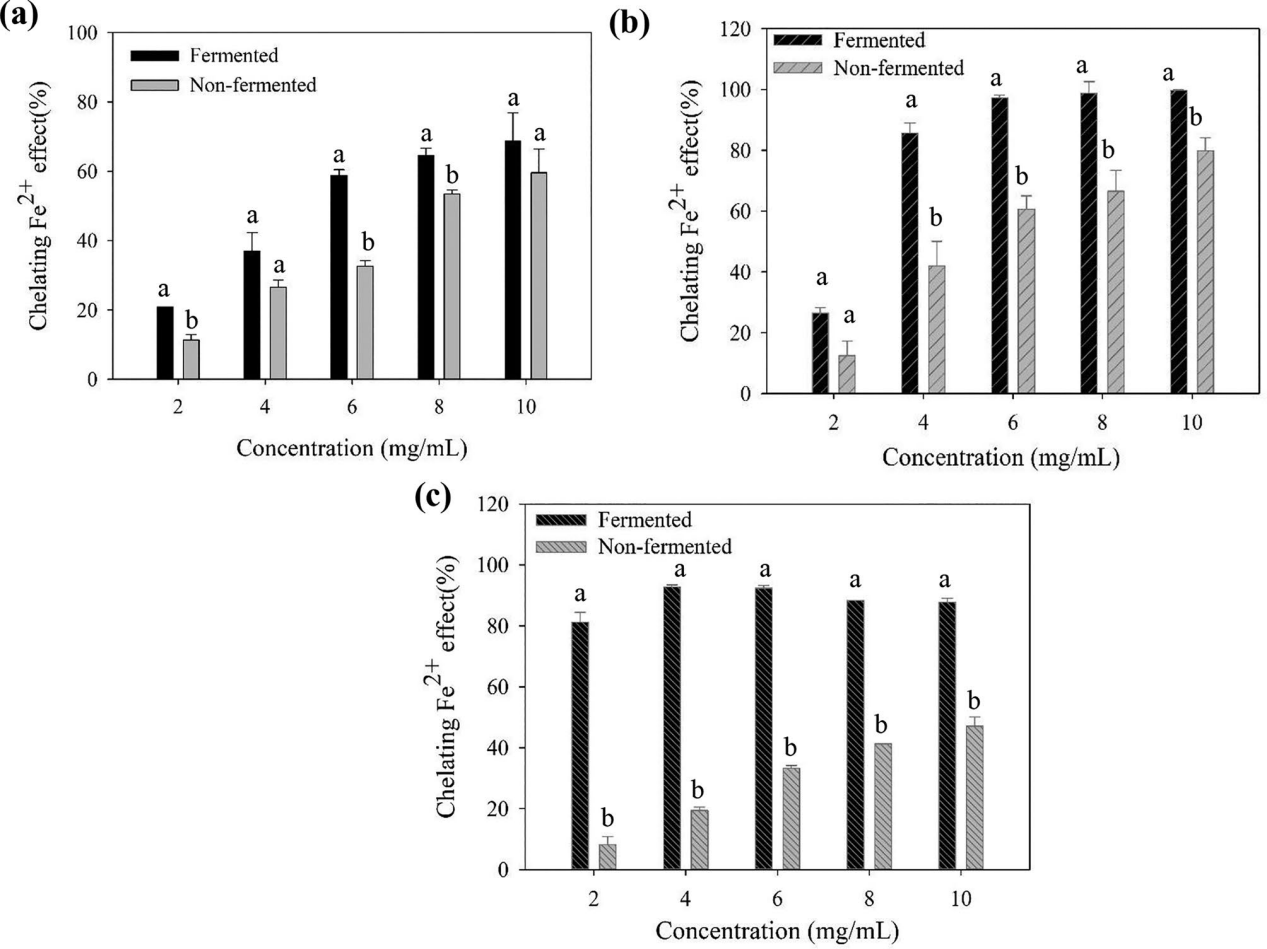
The chelating Fe^2+^ effect of (**a**) supernatants, (**b**) polysaccharides, and (**c**) polyphenols of fermented and non-fermented *L. japonica* at different concentrations. Different letters in each concentration mean significant difference (*p* < 0.05)

Figure 2 demonstrates the ferrous iron-chelating activity of the supernatant (2a), polysaccharide (2b), and polyphenol (2c) fractions from the fermented and nonfermented seaweeds. The results indicated that fermentation could improve the ferrous iron-chelating activity in the supernatant, polysaccharide, or polyphenol fractions, as compared with the nonfermented samples. The fermented or nonfermented supernatants, polysaccharide, or polyphenol fractions showed a concentration-dependent effect on the ferrous iron-chelating activity (Fig. 2a–c). The chelating effect was measured as the decrease in the absorbance of the ferrous/ferrozine complex. In the polysaccharide fraction (Fig. 2b), 4 mg/mL of the fermented seaweed displayed over 86% of the ferrous iron-chelating effect, whereas 42% of the chelating effect was exhibited by the nonfermented sample (4 mg/mL). Sulfated polysaccharide is a potential group from seaweeds, which can chelate ferrous iron in this antioxidant assessment (Costa et al. 2010). Through fermentation, considerable amount of total sugar was released from the brown seaweed, and more sulfate groups were detected in the polysaccharide fraction. The compositional changes caused by fermentation may enhance the ferrous iron-chelating activity compared with the nonfermented seaweed. In addition, hydrophilic phenols extracted from brown seaweeds can serve as potential ferrous iron chelators (Senevirathne et al. 2006). In our polyphenol fraction (Fig. 2c), 4 mg/mL of the fermented seaweed reached the highest chelating effect at 93%, whereas only 19% chelating effect was achieved by the nonfermented sample (4 mg/mL). The number and position of hydroxyl groups are important to the metal-chelating effect. For example, hydroxyl substitution in the *ortho* position promotes the metal-chelating effect (Yen et al. 2000). According to Andjelković et al. (2006), phenolic compounds without a catechol or galloyl moiety (such as vanillic acid, syringic acid, and ferulic acid) showed no ferrous iron-chelating effect, whereas those with catechol or galloyl groups (such as gallic acid, caffeic acid, chlorogenic acid) exhibited strong chelating activity. After fermentation, more phenolic compounds were found in the *L. japonica* suspension (Table 2). Crude polyphenol was extracted from the fermented seaweed, and it showed a higher ferrous iron-chelating effect than the control. Thus, the composition of polyphenols extracted from fermented *L. japonica* and its potential mechanisms on the metal ion-chelating effect is worthy of further investigation.

### Reducing power

**Fig. 3:**
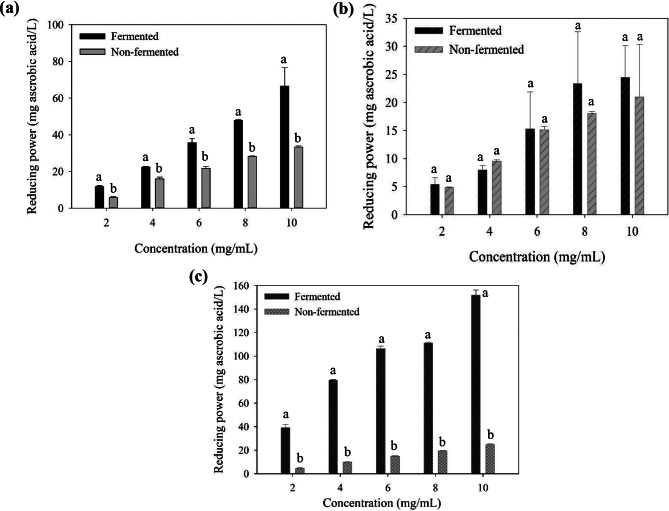
The reducing power of (**a**) supernatants, (**b**) polysaccharides, and (**c**) polyphenols of fermented and non-fermented *L. japonica* at different concentrations. Different letters in each concentration mean significant difference (*p* < 0.05)

Figure 3 shows the reducing power of the supernatant (3a), polysaccharide (3b), and polyphenol (3c) fractions from the fermented and nonfermented seaweeds. These three fractions from either the fermented or the nonfermented seaweed displayed concentration-dependent reducing power. In addition, the supernatant of the fermented *L. japonica* exhibited higher reducing power than that of the nonfermented sample (Fig. 3a), indicating that the bioactive compounds released from the fermented seaweed had a stronger effect on reducing ferricyanide in the assay. As shown in Table 2, fermentation promotes the release of total phenolic compounds, total sugar, and reducing sugar from the seaweed, as compared with the nonfermented group. With regard to the polysaccharide fraction (Fig. 3b), no significant difference was found between the fermented and nonfermented samples under various concentrations. As the reducing power assay measures the capacity of single-electron transfer from the possible antioxidant, the polysaccharide fractions obtained from the fermented or the nonfermented suspension in this study may not contribute to this feature (5–25 mg ascorbic acid/L). The antioxidant with reductone groups (such as ascorbic acid) is more associated with the reducing power assay than the sulfated polysaccharides (Wang et al. 2008). On the contrary, phenolic compounds can donate their electrons through single-electron transfer from the hydroxyl group on the aromatic rings, leading to the antioxidant effect on the reducing power assay. In the polyphenol fraction (Fig. 3c), the fermented samples exhibited stronger reducing power than the nonfermented samples. One significant difference was shown at 10 mg/mL of samples, where fermentation could improve reducing power by approximately six times than the control.

### ABTS radical-scavenging activity

**Fig. 4:**
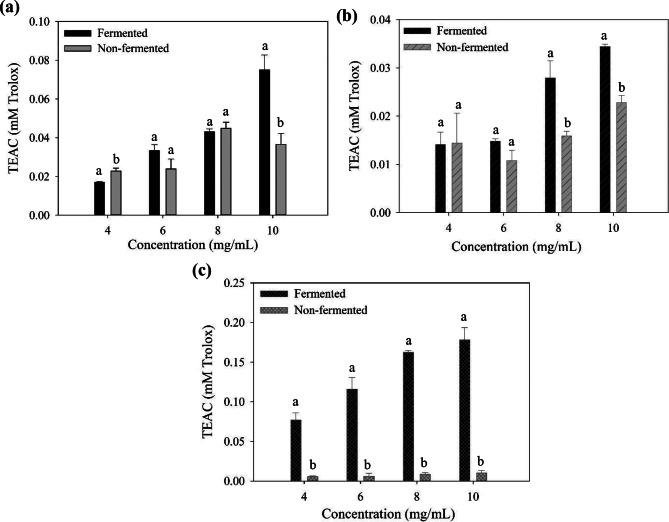
The ABTS^+^ scavenging effect of (**a**) supernatants, (**b**) polysaccharides, and (**c**) polyphenols of fermented and non-fermented *L. japonica* at different concentrations. Different letters in each concentration mean significant difference (*p* < 0.05)

The ABTS-scavenging assay is a widely used method for evaluating the antioxidant capacity of various compounds, including both hydrophilic and lipophilic antioxidants. ABTS-scavenging activity measures antioxidant capacity through the reduction of ABTS radical cations (ABTS•+). When antioxidants react with the blue-green ABTS•+ solution, they convert it back to its colorless neutral form. This color change can be measured spectrophotometrically at 734 nm to quantify antioxidant activity (Nenadis et al. 2004). Figure 4 displays the ABTS radical-scavenging activity of the supernatant (4a), polysaccharide (4b), and polyphenol (4c) fractions from the fermented and nonfermented seaweeds. The concentration dependence of the ABTS radical-scavenging activity was observed in the supernatant (Fig. 4a) and polyphenol (Fig. 4c) fractions from the fermented seaweed. By contrast, the nonfermented samples in all fractions did not show a strong relationship between concentrations and the ABTS radical-scavenging activity. In the supernatant (Fig. 4a) and polysaccharide (Fig. 4b) fractions, the fermented samples at 10 mg/mL represented a higher TEAC value (Trolox equivalent) than the nonfermented sample. On the contrary, no significant difference was found between the two pretreatments at a low sample concentration (6 mg/mL). Although the nonfermented sample expressed higher TEAC in its supernatant at 4 mg/mL than the fermented sample, the supernatants of the fermented or nonfermented seaweed may contain varying levels of bioactive compounds, which contribute to the differences in their antioxidant activity on the ABTS radical-scavenging effect at different sample concentrations. Based on the principle of the ABTS assay, the ABTS radical cation (ABTS•+) can be neutralized through single-electron transfer and hydrogen atom transfer (Re et al. 1999). This process allows the ABTS assay to have broad reactivity with various antioxidants, including hydrophilic and lipophilic compounds (Re et al. 1999). Overall, the synergistic effect of different bioactive compounds in the ABTS assay are more complex than that in other specific antioxidant assays, leading to a non-linear effect on the antioxidant activity. Notably, the polyphenol fraction of the fermented seaweed exhibited stronger ABTS radical-scavenging activity than that of the nonfermented seaweed across all concentrations (Fig. 4c). Based on our findings in the ABTS assay, the purification and characteristics (such as hydrophobicity) of the polyphenol fraction from the fermented seaweed must be further investigated to comprehensively understand the functional properties of polyphenols extracted from fermented *L. japonica*.

### DPPH radical-scavenging activity

**Fig. 5:**
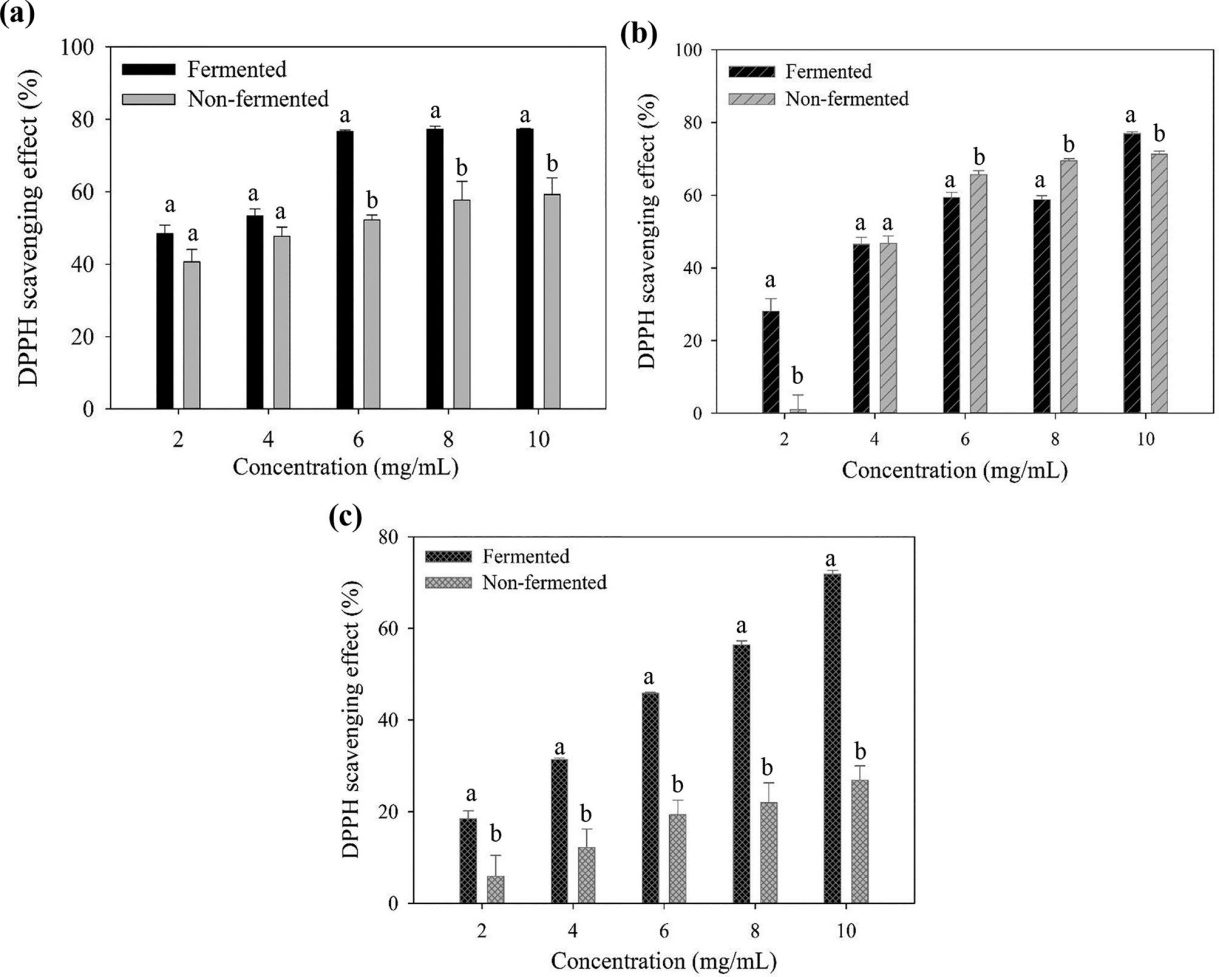
The DPPH radical-scavenging effect of (**a**) supernatants, (**b**) polysaccharides, and (**c**) polyphenols of fermented and non-fermented *L. japonica* at different concentrations. Different letters in each concentration mean significant difference (*p* < 0.05)

Figure 5 displays the DPPH radical-scavenging activity of the supernatant (5a), polysaccharide (5b), and polyphenol (5c) fractions from the fermented seaweed and the nonfermented seaweed. The DPPH radical-scavenging activity of each fraction from the fermented and nonfermented seaweeds increased with the increase of sample concentration. The concentration dependence of the DPPH radical-scavenging activity was observed in the polyphenol fractions of both treatments (Fig. 5c). In general, the supernatant and polyphenol fractions of the fermented seaweed demonstrated greater DPPH radical-scavenging activity than those of the nonfermented samples (Fig. 5a and c). On the contrary, antioxidant responses among different concentrations from the polysaccharide fraction of the fermented and nonfermented seaweeds varied (Fig. 5b). The measurement of the DPPH assay is associated with single-electron transfer and hydrogen atom transfer; however, the scavenging responses vary by different experimental settings, such as the polarity of the test environment (Foti 2015). As fermentation plays an important role in bioconversion, the composition of the polysaccharide fractions from the fermented and nonfermented seaweeds may contribute to the DPPH radical-scavenging activity in a non-linear response. The solubility and diffusion of the antioxidant in the DPPH assay are potential variables of the radical-scavenging effect. In addition, according to Gulcin and Alwasel (2023), the steric hinderance of the antioxidants can affect the DPPH reaction rates. Hence, investigating the structure of the polysaccharide fraction from the fermented seaweed and comparing it with the nonfermented seaweed is necessary. This technique can further clarify the antioxidant potential of the bioactive polysaccharide from the fermented seaweed.

Overall, fermentation can improve the antioxidant activities of the brown seaweed *L. japonica* compared with the nonfermented treatment, in accordance with several antioxidant assays (ferrous iron-chelating activity, reducing power, ABTS radical-scavenging activity, and DPPH radical-scavenging activity). The biological activities of phenolic compounds and sulfated polysaccharides obtained from seaweeds have also been reported (Holdt and Kraan 2011). To enhance the applicability of the bioactive compounds derived from seaweed, fermentation can serve as a promising tool for positive bioconversions. For example, the profiles of phenolic compounds, amino acids, and volatile compounds in the red seaweed extract (*Kappaphycus* spp.) were changed by fermentation, and caffeic acid, as a potential antioxidant, was observed after fermentation (Norakma et al. 2022). The biological activity of seaweed could be enhanced because of microbial bioconversions. Moreover, the antioxidant and anticoagulant properties of the brown seaweed *Sargassum* sp. were enhanced by fermentation with lactic acid bacteria (Shobharani et al. 2013). The fermented *L. japonica* exhibited stronger inhibitory effects on low-density lipoprotein oxidation and foam cell formation compared to the water extract (Lin et al. 2024), which are positive biological activities to reduce the risk of atherosclerosis. In this study, the antioxidant properties of the brown seaweed *L. japonica* were improved through fermentation with *B. subtilis* natto.

The variations in the antioxidant responses can arise from several variables, such as the composition, structure, and conformation of the antioxidants. Farvin and Jacobsen (2013) reported the composition of phenolic compounds from 16 seaweeds, and all water extracts contained gallic and chlorogenic acids. These two phenolic acids are known antioxidants in various foods (Qi et al. 2023). The number and position of hydroxyl groups play an important role in the antioxidant effect of polyphenols. For example, Yen et al. (2000) reported that hydroxyl substitution in the *ortho* position had a positive impact on the metal-chelating effect. With regard to bioactive sulfated polysaccharides, the molecular weight, sulfate content, and monosaccharide composition are known variables for antioxidant activity (Li et al. 2022). However, the antioxidant activity of bioactive compounds from seaweeds is often exhibited by synergistic effects, making it essential to evaluate their activity in real food systems (Farvin and Jacobsen 2013). In the real food system, the sensory characteristics of seaweed-derived products are important for consumer acceptance (Vilar et al. 2020). Apart from enhancing biological activities, fermentation could remove off-flavor compounds through microbial conversions (Hung et al. 2023; Wang et al. 2022). Considering that fermentation can improve the antioxidant activity of the brown seaweed *L. japonica*, future studies on the aromatic profiles of the fermented seaweed will be valuable to shorten or address the limitations in developing commercial seaweed-derived products.

## Conclusions

In the present study, fermentation with *B. subtilis* natto could facilitate the extraction of bioactive compounds from the brown seaweed *Laminaria japonica* and further improve its antioxidant activity. The isolate B2 strain (*B. subtilis* natto) exhibited stronger hydrolytic abilities on fibrin, cellulose, and alginate than other isolates, demonstrating its application potential in effectively degrading seaweed cell wall polysaccharides. After fermentation, the content of phenolic compounds, total sugars, and reducing sugars increased. A higher sulfate content in the polysaccharide fraction of the fermented samples was detected. These results indicate the positive bioconversion achieved by fermentation. In addition, fermentation enhanced the antioxidant activities of the supernatant, polysaccharide, and polyphenol fractions compared with the nonfermented samples. Further research into the composition profile of the bioactive compounds and the aromatic profile of fermented *L. japonica* can promote the development of valuable seaweed-derived products. Through fermentation, seaweed can be processed into various nutraceutical ingredients, making it a potential candidate for food industry applications.

## Data Availability

The datasets used and/or analyzed during the current study are available from the corresponding author on reasonable request.
